# Intraindividual Doppler Flow Response to Exercise Differs Between Symptomatic and Asymptomatic Achilles Tendons

**DOI:** 10.3389/fphys.2021.617497

**Published:** 2021-07-06

**Authors:** Lucie Risch, Josefine Stoll, Anne Schomöller, Tilman Engel, Frank Mayer, Michael Cassel

**Affiliations:** ^1^University Outpatient Clinic, Sports Medicine and Orthopedics, University of Potsdam, Potsdam, Germany; ^2^Faculty of Health Sciences Brandenburg, University of Potsdam, Potsdam, Germany

**Keywords:** Achilles tendinopathy, tendinosis, neovascularization, ultrasound, Advanced Dynamic Flow, sonography

## Abstract

**Objective:**

This study investigated intraindividual differences of intratendinous blood flow (IBF) in response to running exercise in participants with Achilles tendinopathy.

**Design:**

This is a cross-sectional study.

**Setting:**

The study was conducted at the University Outpatient Clinic.

**Participants:**

Sonographic detectable intratendinous blood flow was examined in symptomatic and contralateral asymptomatic Achilles tendons of 19 participants (42 ± 13 years, 178 ± 10 cm, 76 ± 12 kg, VISA-A 75 ± 16) with clinically diagnosed unilateral Achilles tendinopathy and sonographic evident tendinosis.

**Intervention:**

IBF was assessed using Doppler ultrasound “Advanced Dynamic Flow” before (Upre) and 5, 30, 60, and 120 min (U5–U120) after a standardized submaximal constant load run.

**Main Outcome Measure:**

IBF was quantified by counting the number (*n*) of vessels in each tendon.

**Results:**

At Upre, IBF was higher in symptomatic compared with asymptomatic tendons [mean 6.3 (95% CI: 2.8–9.9) and 1.7 (0.4–2.9), *p* < 0.01]. Overall, 63% of symptomatic and 47% of asymptomatic Achilles tendons responded to exercise, whereas 16 and 11% showed persisting IBF and 21 and 42% remained avascular throughout the investigation. At U5, IBF increased in both symptomatic and asymptomatic tendons [difference to baseline: 2.4 (0.3–4.5) and 0.9 (0.5–1.4), *p* = 0.05]. At U30 to U120, IBF was still increased in symptomatic but not in asymptomatic tendons [mean difference to baseline: 1.9 (0.8–2.9) and 0.1 (-0.9 to 1.2), *p* < 0.01].

**Conclusion:**

Irrespective of pathology, 47–63% of Achilles tendons responded to exercise with an immediate acute physiological IBF increase by an average of one to two vessels (“responders”). A higher amount of baseline IBF (approximately five vessels) and a prolonged exercise-induced IBF response found in symptomatic ATs indicate a pain-associated altered intratendinous “neovascularization.”

## Introduction

Midportion Achilles tendinopathy is a frequent diagnosis in running and jumping athletes (Hirschmüller et al., 2010) but is also found in the general population (de Jonge et al., 2011). It is characterized by tendon pain, local swelling, and morning stiffness and often results in impaired performance (Maffulli et al., 1998; van Dijk et al., 2011; Cook et al., 2016). The clinical diagnosis can be based on a positive history of tendon pain combined with pain on palpation of the tendon (Hutchison et al., 2013). A sonographic examination additionally enables the evaluation of degenerative tissue alterations (tendinosis) visible as spindle-shaped thickening or hypo/hyperechogenic areas (Hirschmüller et al., 2012). The diagnostic value of intratendinous blood flow (IBF) detectable with Doppler ultrasound, however, is controversially discussed (Peers et al., 2003; Yang et al., 2010; Tol et al., 2012).

IBF has been detected in up to 88% of Achilles tendinopathy patients (Tol et al., 2012) and has been associated with the ingrowth of neo-innervation responsible for the onset of Achilles tendon pain in the context of a failed healing response (Cook and Purdam, 2009). However, cross-sectional as well as prospective studies have reported no clear relation between the amount of IBF and pain, functional impairment, or treatment outcome (Malliaras et al., 2008, 2013; Boesen et al., 2012). Moreover, the detectability of a low amount of IBF in up to 35% of asymptomatic Achilles tendons has led researchers to question its mere association with pain and pathology (Boesen et al., 2006b; Hirschmüller et al., 2010). A threshold to distinguish between occurrence of low physiological (one to two vessels) and high pathological IBF amount (> 2 vessels) has been proposed but, so far, lacks evidence (Boesen et al., 2012, 2006b). In athletes, a frequent occurrence of IBF at rest as well as an exercise-induced IBF increase detected directly following competition has been considered to indicate a long-term adaptation to mechanical loading (Malliaras et al., 2008) and an acute physiological response due to increased metabolic demands (Boesen et al., 2006a,b; Malliaras et al., 2008). In patients with Achilles tendinopathy, exercise-induced IBF increase has been reported in a majority of tendons following eccentric exercise. In asymptomatic Achilles tendons, running had a similar effect (Boesen et al., 2006b). Overall, it remains questionable whether sonographic detectable IBF is a physiological finding or is associated with pain and pathology. Therefore, the aim of this study was to investigate intraindividual differences of Achilles tendon IBF before and after a standardized running exercise in patients with unilateral Achilles tendinopathy, comparing the symptomatic and contralateral asymptomatic side.

## Materials and Methods

**FIGURE 1:**
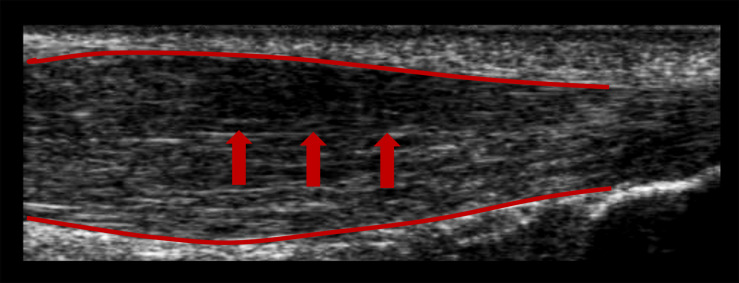
B-mode ultrasound image showing a hypoechogenic area (arrows) and spindle-shaped thickening (red lines) of a symptomatic Achilles tendon.

Nineteen patients (3 females/16 males, 42 ± 13 years, 178 ± 10 cm, 76 ± 12 kg) with clinically diagnosed unilateral midportion Achilles tendinopathy combined with sonographically confirmed presence of structural alterations (tendinosis) were included in this cross-sectional study after providing written informed consent. Since training level does not influence exercise-induced IBF (Risch et al., 2021), participants were included irrespective of their amount of habitual physical activity. Exclusion criteria were any known systemic diseases (e.g., cardiovascular, metabolic, or rheumatic disease), prior partial or complete Achilles tendon ruptures, bilateral tendinopathy, or a history of tendon pain in the currently unaffected limb, as well as injections or surgical interventions at the Achilles tendon. Physiotherapy/physical therapy treatment was not considered. Appropriate inclusion was ensured by a medical examination performed by a sports medicine physician. The criteria for the presence of tendinopathy were history of Achilles tendon pain as well as pain on palpation of the tendon (Hutchison et al., 2013). Tendinotic tissue alterations were defined as sonographically detectable hypoechogenicity and focal thickening of the tendon (Figure 1; Hirschmüller et al., 2012; Hutchison et al., 2013). Furthermore, all participants filled out the Victorian Institute of Sports Assessment-Achilles questionnaire to quantify pain and functional impairment (VISA-A score from 0 to 100; lower score referring to more pain/impairment; Lohrer and Nauck, 2009).

The study consisted of two measurement days (M1, M2) separated by a minimum of 2 days. On M1, all participants performed a maximum incremental treadmill running exercise test with lactate and heart rate measurements to determine the individual anaerobic threshold. On M2, participants performed a 30-min constant load treadmill running exercise (additional 9 min warm-up with incremental increase of velocity to target speed; lactate and heart rate measurement during constant load running every 10 min to control for steady state) with the velocity set to 5% below the individual anaerobic threshold. All running tasks were performed shod and on the same treadmill (Pulsar, h/p/cosmos Sports & Medical, Nussdorf-Traunstein, Germany) with a constant incline of 0.4% (Mugele et al., 2018). Doppler ultrasound examinations were conducted before (Upre) constant load running and 5, 30, 60, and 120 min (U5–U120) afterward. All participants were asked to abstain from physical activity 24 h prior to the measurement to eliminate any effect of exercise on the baseline ultrasound examination. In between postexercise ultrasound examinations, participants were asked to stay passive. Applicability and high reliability of the running exercise and ultrasound examination protocol have already been reported previously (Risch et al., 2018a). The study was approved by the local ethics committee.

**FIGURE 2:**
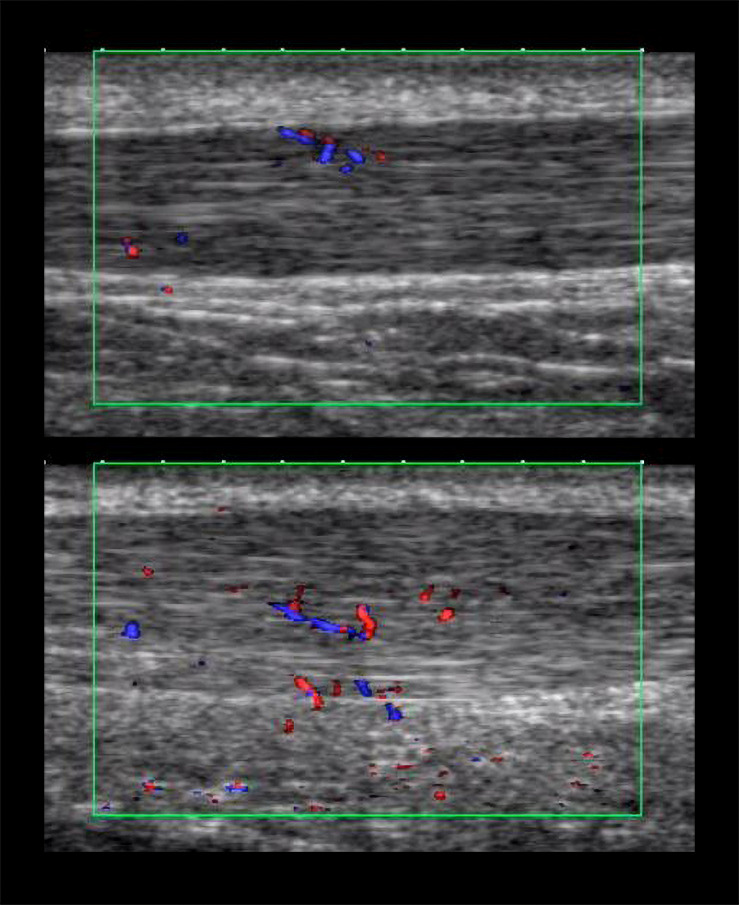
Momentary images from intratendinous blood flow (IBF) video sequences of an asymptomatic **(top)** and symptomatic **(bottom)** Achilles tendon.

All examinations were performed by the same observer (2 years of regular practice, not blinded to the participant) with the same high-resolution ultrasound device (Xario SSA-660 A, Toshiba, Japan) using a multifrequency linear transducer at 14 MHz (PLT-120 AT). Achilles tendons (ATs) were examined in prone position with the participants’ feet hanging over the distal end of the examination table. Baseline examinations on M1 included longitudinal and transverse B-mode ultrasound scans (gain = 80, *DR* = 65, penetration depth = 3 cm, focus = 0.5 cm) to assess hypoechoic tissue alterations as well as maximal anterior–posterior tendon thickness in the midportion with the ankle passively placed at 90° angle to tibia. On M2, IBF was assessed throughout the tendon (from calcaneal insertion to musculotendinous junction, excluding the paratenon/peritendinous tissue) with the feet hanging free to avoid obliteration of vessels, performing longitudinal recordings using the broadband color DU “advanced dynamic flow” (box region of interest 2 × 1 cm, presettings: color gain 42, color velocity 1.5 cm/s, pulse repetition frequency 13.7 kHz) saving video sequences of 5 s duration (Risch et al., 2016, 2018a; Figure 2). The order of the left and right tendon examination was randomized (irrespective of the site of pain) for each measurement time point. IBF was quantified using the counting score which has shown to be an easily applicable and reliable quantification method (Risch et al., 2018b). For this score, the number of vessels (*n*) throughout each Achilles tendon is determined by counting each vessel branch as one vessel (Risch et al., 2018b).

Anthropometric data and tendon characteristics were analyzed descriptively with mean ± SD, percentage, and minimum to maximum range. Normal distribution of data was checked with the Shapiro–Wilk test. Side differences regarding tendon thickness were analyzed with the Student’s *t*-test. Intraindividual differences in IBF between the number of baseline vessels in the symptomatic and contralateral asymptomatic ATs are presented in absolute values. Changes of IBF over time are presented as mean difference (delta) to baseline. Side differences of absolute baseline IBF, delta change from Upre to U5, and mean delta change from Upre to U30 to U120 are presented with mean and 95% confidence interval (CI). Statistical significance of side differences was tested with the Wilcoxon signed rank test. A minimum change of two vessels following exercise was considered a responder according to a standard error of measurement between 0.99 and 1.47 (Risch et al., 2018b). Association between baseline VISA-A score (total score) and IBF in the symptomatic tendon at each measurement time point was determined with the Spearman’s rho correlation coefficient. Alpha-level was set to < 0.05.

## Results

**FIGURE 3:**
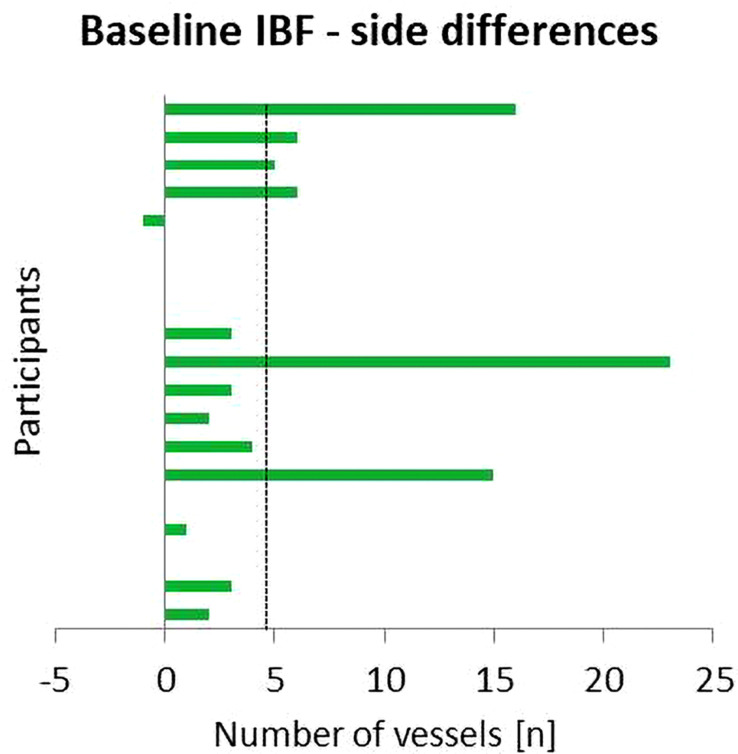
Side differences between IBF at baseline (Upre) in the symptomatic and asymptomatic Achilles tendon (AT). Positive values indicate higher amount of IBF in the symptomatic AT; dashed line indicates mean difference.

From the 25 recruited participants with Achilles tendinopathy, one participant was excluded due to a cardiovascular disease, four participants were excluded due to a history of pain in both Achilles tendons, and one participant dropped out of the study after M1 due to an intense onset of Achilles tendon pain. The 19 participants with unilateral Achilles tendinopathy included in this analysis reported a VISA-A score of 75 ± 16 (range 60–94). The duration of tendon pain ranged from 2 months to 4 years. All participants were in a state of subacute or chronic pain enabling a continuation of training/activity. Regular physical activity ranged from 1 up to 40 h per week including running, fitness, cycling, and swimming. The mean running velocity during the constant load exercise (M2) was 11.5 ± 2.1 km/h. Tendon anterior–posterior thickness was higher in symptomatic compared with asymptomatic ATs (6.7 ± 1.3 vs. 5.9 ± 1.1 mm, *p* < 0.01). Hypoechogenic areas and focal tendon thickening were visible in all symptomatic ATs, whereas hypoechogenicity was also detected in eight and focal thickening in three asymptomatic ATs (Table 1). In 13 participants, the amount of IBF was higher in the symptomatic compared with the asymptomatic AT with a mean difference of 4.6 vessels (95% CI 1.5–7.7) (Figure 3).

**TABLE 1:** Tendon characteristics of the symptomatic and asymptomatic side.

	Symptomatic side (*n* = 19)	Asymptomatic side (*n* = 19)
Spindle-shaped thickening	19/19(100%)	3/19(16%)
Hypoechogenicity	19/19(100%)	8/19(42%)
IBF (Upre)	14/19(74%)	8/19(42%)
IBF (Upre) and structural alterations	14/19(74%)	5/19(26%)
IBF responders	12/19(63%)	9/19(47%)
IBF non-responders	7/19(37%)	10/19(53%)
–With persisting IBF	3/19(16%)	2/19(11%)
–With no IBF at all	4/19(21%)	8/19(42%)

**FIGURE 4:**
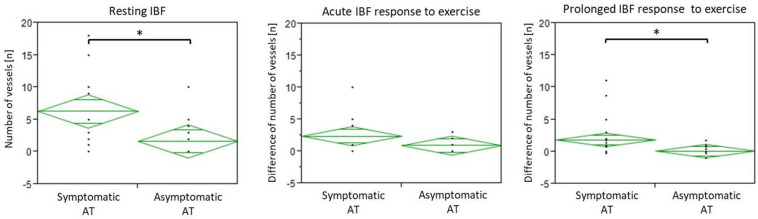
Diamond plots showing mean and 95% CI of IBF at baseline (resting IBF), difference between U5 and Upre (acute response), and mean difference between U30–120 and Upre (prolonged response). ^∗^Indicates significant intraindividual differences (*p* < 0.01).

**FIGURE 5:**
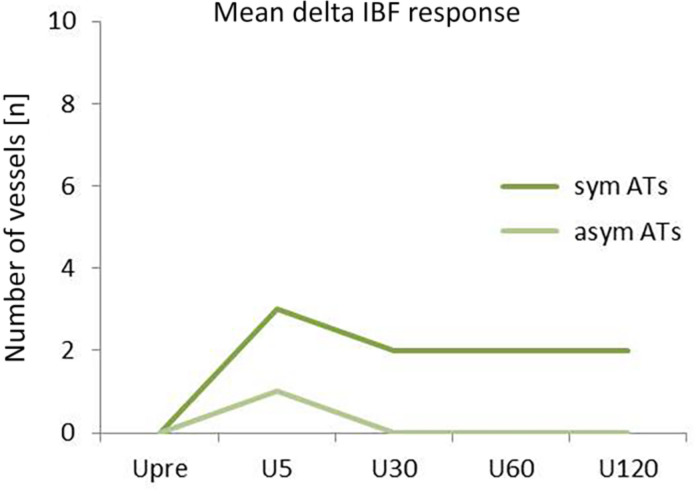
Mean IBF changes in response to exercise (difference to baseline) for symptomatic (sym) and asymptomatic (asym) ATs over the course of time (Upre–U120).

**FIGURE 6:**
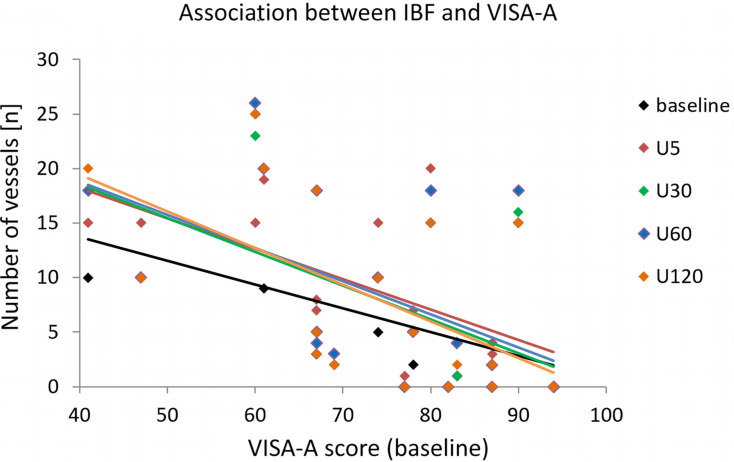
Scatterplot with trend lines indicating the association between the number of vessels in symptomatic tendons for each measurement time point and the VISA-A score assessed at baseline.

IBF side differences are presented in Figures 3–5. At baseline, IBF was detectable in 74% (14/19) of symptomatic ATs [whole group: mean 6.3 (95% CI: 2.8–9.9), absolute range from 0 to 25 vessels] and in 42% (8/19) of asymptomatic ATs [whole group: 1.7 (0.4–2.9), absolute range from 0 to 10 vessels]. Side differences were statistically significant (*p* < 0.01, Figure 4). At U5, IBF responded to exercise in 63% (12/19) of symptomatic ATs with a difference to baseline of 2.4 vessels [(0.3–4.5), absolute range from 0 to 20 vessels]. In asymptomatic ATs, 47% (9/19) responded to exercise at U5 with a mean IBF increase of 0.9 [(0.5–1.4), absolute range from 0 to 12 vessels]. The amount of increase from baseline to U5 was not significantly different between the symptomatic and asymptomatic ATs (*p* = 0.05, Figures 4, 5). In 10 from 12 responding symptomatic AT, IBF remained elevated up to U120 with a mean difference to baseline of 1.9 (0.8–2.9) vessels. In seven from nine responding asymptomatic tendons, exercise-induced IBF increase returned to baseline values at U30–U120, with a mean difference to baseline of 0.1 (-0.9 to 1.2) vessels. Mean IBF difference to baseline at U30–U120 was significantly higher for the symptomatic compared with the asymptomatic tendon (*p* < 0.01, Figures 4, 5). While 16% (3/19) of all symptomatic and 11% (2/19) of all asymptomatic ATs showed no changes of IBF, 21% (4/19) and 42% (8/19), respectively, remained without detectable IBF throughout the investigation. A higher IBF amount in symptomatic ATs measured before as well as after exercise was negatively correlated to the VISA-A score (*r* = -0.61 to -0.67, *p* < 0.01, Figure 6).

## Discussion

The aim of this study was to investigate intraindividual differences of IBF occurrence in response to running exercise in patients with unilateral Achilles tendinopathy comparing the symptomatic and asymptomatic side. At baseline, IBF was detected more frequently in symptomatic tendons with an average amount of six vessels compared with asymptomatic tendons with an average amount of two vessels. The immediate effect of acute submaximal running exercise on IBF did not differ with respect to tendon pain: 47–63% of Achilles tendons were “responders,” reacting with an increase of one to two vessels detectable directly after exercise, whereas 37–53% of Achilles tendons were “non-responders,” showing no change of IBF or no IBF at all. In responding asymptomatic Achilles tendons, exercise-induced IBF increase returned to baseline level after 30 min, whereas exercise-induced IBF increase in responding symptomatic ATs continued to be detectable up to 120 min after running.

In line with the literature, symptomatic Achilles tendons revealed a more frequent occurrence and higher amount of (resting) IBF compared with asymptomatic Achilles tendons (Zanetti et al., 2003; Reiter and Ulreich, 2004; Boesen et al., 2006b; Sengkerij et al., 2009). Previous studies have found IBF in 50–88% of tendinopathic Achilles tendons (Peers et al., 2003; Zanetti et al., 2003; Reiter and Ulreich, 2004; de Vos et al., 2007) but only in 4–35% of asymptomatic controls (Zanetti et al., 2003; Boesen et al., 2006b; Sengkerij et al., 2009; Hirschmüller et al., 2010). The amount of IBF has been rated with various scoring procedures, consistently reporting a lower amount in asymptomatic tendons (Zanetti et al., 2003; Reiter and Ulreich, 2004; Boesen et al., 2006b; Sengkerij et al., 2009). The present findings comparing intraindividual differences of IBF between the symptomatic and contralateral asymptomatic tendon, furthermore, support the assumption that altered IBF is a local finding related to tendon pain. Various studies have suggested IBF to be associated with ingrowth of neo-innervation responsible for the onset of symptoms (Ohberg and Alfredson, 2002; Alfredson et al., 2003; Andersson et al., 2007; Tol et al., 2012). The continuum model of tendon pathology introduced by Cook and Purdam (2009) has described IBF occurrence especially in the stages of tendon disrepair and degeneration with severe tissue turnover in chronically overloaded tendons. Although Doppler examinations have shown increased blood flow in symptomatic degenerated Achilles tendons (Ohberg et al., 2001; Alfredson et al., 2003; Boesen et al., 2006b), simultaneously observed high levels of lactate (at rest) have suggested a hypoxic condition negatively affecting tissue regeneration (Alfredson et al., 2002; Järvinen, 2020). It has been hypothesized that “neovascularization” occurring due to the hypoxia-induced expression of vascular–endothelial growth factors consists of non-functional, hyperpermeable vessels that fail to supply the tendon tissue that is attempting to heal (Tol et al., 2012; Järvinen, 2020). In this context, elevated resting IBF found in symptomatic degenerated Achilles tendons compared with the contralateral asymptomatic Achilles tendon in the present study can be rated as neovascularization associated with tendon pain and pathology. The moderate association between the amount of IBF and pain severity/functional impairment assessed by the VISA-A questionnaire (Lohrer and Nauck, 2009), moreover, supports a quantitative relationship between the degree of IBF and pain level also reported by Reiter and Ulreich (2004).

In contrast to the hypothesis of a mere association between IBF and the onset of tendon pain (Ohberg and Alfredson, 2002), the present investigation found IBF in 42% of contralateral asymptomatic Achilles tendons presenting up to 10 vessels at rest. Sonographic detectable IBF in up to 35% of healthy Achilles tendons has been assumed to represent a physiological occurrence due to increasing sensitivity of ultrasound devices to low blood flow (Boesen et al., 2006b, 2012; Tol et al., 2012). Boesen et al. (2012) have suggested a threshold to differentiate between the low amount of physiological IBF (one to two vessels) and the higher amount of pathological IBF (> 2 vessels). However, this threshold lacks evidence and seems too low in light of the present findings. The use of the high-sensitive Doppler mode “advanced dynamic flow” with a wider frequency range and higher frame rate resulting in improved resolution and a more precise visualization of IBF compared with conventional Doppler modes (Heling et al., 2004; Risch et al., 2016) has enabled a very detailed, reliable quantification and monitoring of vessels with the use of the counting score (Risch et al., 2018b). The clear differentiation of neighboring vessels due to less overpainting of vessel walls has most likely resulted in a higher number of detected vessels. Therefore, the definition of a threshold seems to depend on the technical quality and setting of the device and, furthermore, requires an adequate, sensitive scoring procedure (Boesen et al., 2006b, 2012; Risch et al., 2018b).

What should be taken into consideration when interpreting the present data in comparison with previous studies on healthy controls is that the majority of asymptomatic Achilles tendons with IBF also revealed structural alterations. It is known from previous literature that the unaffected tendon in patients with tendinopathy is frequently involved in asymptomatic tendinosis (Richards et al., 2005) and previous studies have associated structural alterations with the occurrence of IBF (Ohberg et al., 2001; Zanetti et al., 2003; Malliaras et al., 2008). Consequently, these asymptomatic tendons cannot be considered as “healthy,” inconspicuous control tendons, and it can be argued that the presence of IBF could also represent early tissue degeneration (Fahlström and Alfredson, 2010; Cassel et al., 2015) or a predisposition to tendon pathology (Hirschmüller et al., 2012). Overall, the diagnostic relevance of sonographic detectable IBF in the absence of pain remains yet to be clarified.

Submaximal running exercise resulted in an immediate increase of approximately one to two vessels in both symptomatic and asymptomatic Achilles tendons, supporting the theory of an acute physiological reaction to tendon loading activity, irrespective of pain and pathology (Boesen et al., 2006a,b; Risch et al., 2018a). Although previous studies have also reported an increase of IBF following exercise in tendinopathy patients as well as in healthy athletes and non-athletes (Boesen et al., 2006a,b; Fahlström and Alfredson, 2010; Pingel et al., 2013a,b; Risch et al., 2018a), comparability to the present data is limited due to varying exercise and examination procedures. Interestingly, only 47–63% of Achilles tendons showed an increase of IBF following exercise and are considered “responders,” whereas 37–53% of Achilles tendons were “non-responders” without IBF changes. Since this condition occurred in both symptomatic and asymptomatic tendons, an association to pain or pathology seems unlikely. Nevertheless, it remains unclear why exercise-induced IBF appears in approximately half of the tendons, whereas tendon perfusion in the other half remains below the sensitivity threshold of ultrasound devices (Pingel et al., 2013a,b).

In 80% of responding asymptomatic tendons in the present study, increased IBF was solely detectable 5 min after running which is comparable with the vasodilatory effect with exercise resulting in increased perfusion seen in muscle tissue (Hellsten et al., 2012). Intratendinous blood flow is suggested to temporarily increase above the sensitivity threshold of ultrasound devices thereby appearing in Doppler examinations before decreasing and becoming invisible again after 30 min. In 85% of responding symptomatic Achilles tendons, however, the acute increase following exercise remained detectable up to 120 min, suggesting altered vascularization which seems to be associated with pain. It is hypothesized that this prolonged elevation of exercise-induced IBF is a consequence of pathological, dysfunctional “neovascularization” (Cook et al., 2005; Tol et al., 2012; Järvinen, 2020), in contrast to the physiological short-term increase seen in asymptomatic tendons directly after exercise. Therefore, prolonged detectability of exercise-induced IBF is proposed to be an indicator of tendon pain and pathology, and the examination time point enables a differentiation between physiologically increased IBF, detectable after 5 min, and pathologic increased IBF, detectable after 30 min. A limitation of the study which may influence the generalizability of the presented results is the rather small sample size. Due to a lack of previous data with a similar methodological approach and tendon/patient characteristics, no power analysis was performed. Nevertheless, the sample size is comparable with earlier studies investigating exercise-induced IBF (Boesen et al., 2006b; Malliaras et al., 2012; Pingel et al., 2013a).

In conclusion, a more frequent occurrence (74%) and a higher amount (six vessels on average) of IBF in symptomatic Achilles tendons compared with contralateral asymptomatic Achilles tendons (42%, two vessels) are local pathological findings associated with tendon pain. Irrespective of pain and pathology, exercise results in an immediate IBF increase of one to two vessels which is considered a physiological response. This response can be observed in approximately 50% of all tendons. In 80% of asymptomatic responding Achilles tendons, this physiological exercise-induced increase of IBF returns to baseline after 30 min, whereas 85% of symptomatic responding Achilles tendons show a prolonged exercise-induced increase of IBF detectable up to 120 min after exercise indicating pathological neovascularization.

## Data Availability Statement

The raw data supporting the conclusions of this article will be made available by the authors, without undue reservation.

## Ethics Statement

The studies involving human participants were reviewed and approved by the Ethics Committee University of Potsdam. The patients/participants provided their written informed consent to participate in this study.

## Author Contributions

LR was responsible for developing and performing the study and writing the manuscript. JS, AS, and TE were involved in data interpretation and developing the manuscript. FM and MC were involved in developing the study design, data interpretation, and revising the manuscript. All authors contributed to the article and approved the submitted version.

## Conflict of Interest

The authors declare that the research was conducted in the absence of any commercial or financial relationships that could be construed as a potential conflict of interest.
